# Relation between choice-induced preference change and depression

**DOI:** 10.1371/journal.pone.0180041

**Published:** 2017-06-29

**Authors:** Madoka Miyagi, Makoto Miyatani, Takashi Nakao

**Affiliations:** 1 Graduate School of Education, Hiroshima University, Higashi Hiroshima, Hiroshima, Japan; 2 Department of Psychology, Hiroshima University, Hiroshima, Japan; Centre national de la recherche scientifique, FRANCE

## Abstract

Most experimental studies of depressive symptom effects on decision-making have examined situations in which a single correct answer exists based on external circumstances (externally guided decision-making, e.g., gambling task). In addition to such decision-making, for decision-making of other types, no correct answer exists based on external circumstances (internally guided decision-making, e.g., preference judgment). For internally guided decision-making, a phenomenon is known by which preference for the chosen item increases and preference for the rejected item is decreased after choosing between two equally preferred items which is designated as choice-induced preference change. Recent reports suggest that this phenomenon is explainable by reinforcement learning theory just as it is with externally guided decision-making. Although many earlier studies have revealed the effects of depression in externally guided decision-making, the relation between depressive symptoms and choice-induced preference change remains unclear. This study investigated the relation between depressive symptoms and choice-induced preference change using the blind choice paradigm. Results show that depressive symptoms are correlated with change in preference of rejected items (Spearman’s *r* = .28, *p* = .04): depressed individuals tend to show less decreased preference of rejected items. These results indicate that individual differences of depressive symptoms affect choice-induced preference change. We discuss the mechanisms underlying the relation between depression and choice-induced preference change.

## Introduction

Depression is among the world’s greatest public health problems. The World Health Organization [1] reported that depression affects more than 350 million people of all ages. As reviewed in the literature [2,3], depressive symptoms are well known to affect various aspects of cognitive functions such as memory, attention, and problem-solving. The effects of depression on decision-making constitute a broadly investigated theme of psychology and neuroscience [4–11].

Many studies of depression effects on decision-making have used tasks in which a particularly more or less predictable answer is available, called externally guided decision-making [12,13]. In such situations, people must adjust their choices to comply with an externally defined single correct answer indicated by feedback. The learning process of an externally defined rule is generally explained by reinforcement learning (RL) theory by which the expected value for choosing an option is updated to minimize the difference between the actual and expected value (i.e., prediction error, [14–16]). Depressed individuals are known to show impaired RL in a reward-based decision making task [2,6,9,17]. For example, Pizzagalli, Jahn, & O’Shea [18] used a probabilistic reward task in which participants were asked to identify whether the mouth length of a cartoon face was short or long. After choices, participants were given rewards when the choice was a correct response. Results showed that depressed individuals were less likely to choose highly rewarded alternatives than non-depressed individuals were. Hyposensitivity to reward of the depressed individual is probably the cause of the impaired RL [5,19].

In addition to externally guided decision-making, decision-making also exists for which no correct answer is available for a subject based on external circumstances [12,13,20–26]. Such decision-making is designated as internally guided [12]. These decisions are usually made in the context of preference judgment [13,27–32] and in the context of moral decision-making [33–36]. Internally guided decision-making of the answer depends on the subject's own, i.e. internal, preferences rather than on external, i.e. circumstantial, criteria. No prediction error is available for internally guided decision-making. However, the value or preference of an option is known to be altered through the results of the subject’s own decision. For instance, the preference for the chosen item is increased, although that for the rejected item is decreased. This phenomenon, designated as choice-induced preference change, has been demonstrated using the free-choice paradigm [37]. Typical free-choice paradigms consist of three tasks: pre-choice rating, choice, and post-choice rating. In the pre-choice rating task, a participant rates several items according to the participant’s own preferences. In the choice task, two items are presented. Then a participant is asked to choose the more preferred one. In the post-choice rating phase, a participant is asked to rate all items again in the same manner as that used for pre-ratings. Many earlier reports have described that the items chosen from equally preferred alternatives become more preferred and that the rejected items are less preferred in the post-choice rating task than in the pre-choice rating task [38–41]. Although many earlier studies have examined depression effects on externally guided decision-making, no report in the relevant literature has addressed that effect related to internally guided-decision making.

Recently, an RL-like mechanism is thought to explain how choice-induced preference change occurs [42–44]. Akaishi et al. [42] demonstrated that people tend to repeated the same decision when they are compelled to make a decision based on a perceptually ambiguous stimulus with no performance feedback. Importantly, they showed that the underlying choice repetition is explained well by the RL-based model in which the likelihood of choosing an option is updated by the choice itself. Although Akaishi et al. [42] used a perceptual decision-making task, which was more externally guided than internally guided, they proposed that the same mechanism can explain the choice-induced preference change. Their proposition holds that, for a situation in which no externally defined single correct answer exists, choosing a particular item is followed by reinforcement of the chosen item as if the own choice is regarded as a correct answer. This process engenders an increased preference for the chosen item. Consistent with this explanation, neuroimaging studies using the free-choice paradigm have demonstrated activation of the ventral striatum and ventromedial prefrontal cortex [40,45–47], which is typically associated with reward-based reinforcement learning [16,48,49]. Based on this evidence, it is likely that a common or similar underlying learning mechanism exists between externally guided and internally guided decision-making, and that depression affects the choice-induced preference change just as it does in the case of externally guided decision-making. Nevertheless, no report of the relevant literature has described a study exploring the relation between depression and choice-induced preference change.

This preliminary study investigated the relation between depression and choice-induced preference change. We predict that individuals with strongly depressive symptoms tend to show less choice-induced preference change. As a first step, for this study, we examined depressed participants in a non-clinical sample. Many reports of the literature have described that non-clinically depressed people show similar symptoms in major depressive disorders. Indeed, studies of externally guided decision-making have revealed that depressed participants in a non-clinical sample exhibit difficulty with reinforcement of items according to their own choices, to the same degree as major depressive disorder patients [2,6,9,17]. If depressive symptoms affect the choice-induced preference change, then one would observe a relation between depressive symptoms and choice-induced preference change, even in depressed participants in a non-clinical sample, as in a case with externally guided decision-making.

We used the blind choice paradigm [50] instead of the traditional free-choice paradigm, which has severe methodological difficulties [51,52]. Chen and Risen [51] argued that the traditional free-choice paradigm can produce the predicted preference change with no change in true preference. Reports of the literature explain [51,52] that one cause of this problem is categorization of the items into chosen or rejected items based on participants’ decisions during the choice task. In the blind choice paradigm, the items were categorized randomly, but participants were led to believe that they had chosen the randomly selected item (see Methods for more details about the procedures). Many earlier studies [52–54] have demonstrated that the blind choice paradigm can avoid the problem pointed out by Chen and Risen [51].

## Methods

### Participants

**Fig 1:**
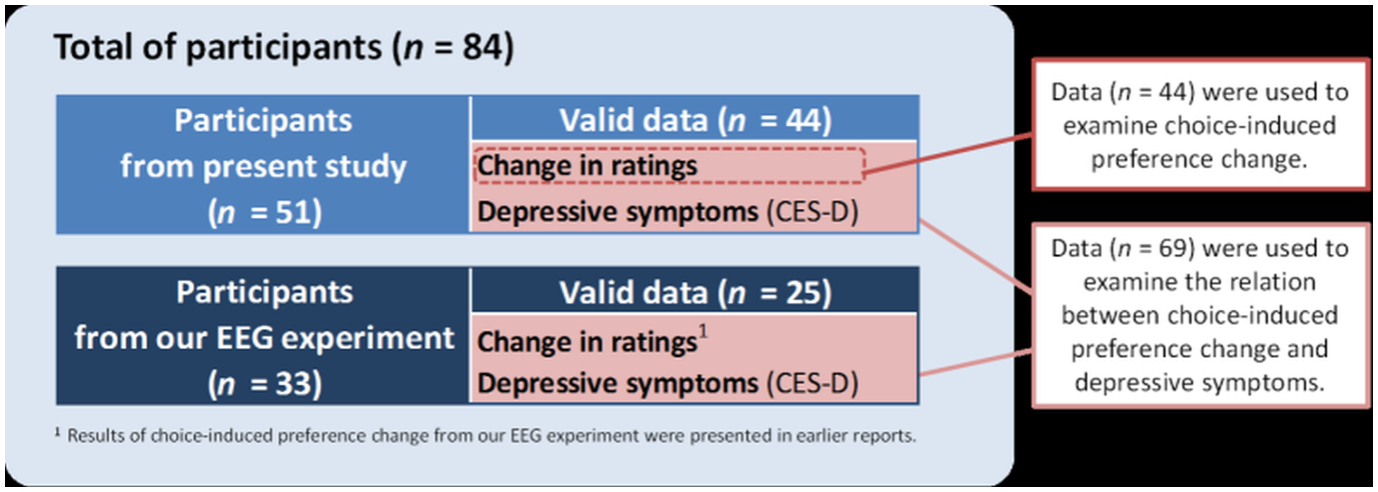
Study participants. We collected 84 participants’ data. Of those data, 33 participants’ data related to change in ratings and depressive symptoms (i.e., CES-D scores) were obtained from earlier EEG experiments conducted using same paradigm as that used for the present study. Data of two participants were excluded because of technical problems with a personal computer (PC). In addition, data of 13 participants were excluded because the participants did not believe the deception of the blind choice task. Results related to choice-induced preference change from our EEG experiment were presented in our previous paper [56]. Therefore, we used the data after excluding our EEG experiment data obtained when ascertaining whether choice-induced preference change was observed. We conducted correlation analysis between the change of preference ratings and CES-D for the sample size of *n* = 69.

In this experiment, 51 Japanese undergraduate and graduate students participated. Furthermore, when we examined the relation between depression and choice-induced preference change, we added data about the change in ratings and CES-D scores (i.e., depressive symptoms) of 33 Japanese undergraduate and graduate students from our earlier EEG experiment using the same paradigm as that of the present study [55]. The total data were those of 84 participants (see Fig 1). Two participants were excluded because of technical problems with a personal computer (PC). Thirteen participants were also excluded because they did not believe the deception of the blind choice task. Consequently, the valid data were those of *n* = 69 participants (40 female, mean age = 19.7, age range = 18–26). The result of choice-induced preference change from our EEG experiment was presented in our previous paper [55]. Therefore, when we examined choice-induced preference change itself, we excluded data from the EEG experiment. For that reason, data of 44 participants were used to examine choice-induced preference change. This report describes a specific examination of the relations between the change in ratings and depressive symptoms, which was not included in our earlier reports. Therefore, data of 69 participants were used for correlation analyses.

All participants had normal or corrected-to-normal vision. No participant was taking any medication. None had a history of neuropsychiatric disorders. The study was approved by the ethics committee of the Graduate School of Education, Hiroshima University. All participants gave written informed consent before the start of the experiment.

### Measures

#### Self-reported depressive symptom level

Each participant’s tendency to depression was assessed using the Japanese version of the Center for Epidemiological Studies Depression Scale (CES-D [56]; Japanese version [57]). Shima et al. demonstrated the validity and reliability of the Japanese version of the CES-D [58]. This scale, which is used for identifying depressive symptoms and depressive disorder in the general population, is often used in psychological studies [6,7]. Participants were asked how often they had felt this way during the prior week. CES-D consists of 20 items (e.g., “*I was bothered by things that usually do not bother me*.”), with responses made using a four-point scale from “*rarely or none of the time*” to “*most or all the time*.” Higher scores indicate a higher level of depressive symptoms.

The average of CES-D scores is 15.1 (*SD* = 8.6, *n* = 69). The score range in our study participants was 2–41. We found no gender difference in CES-D scores (*t*(67) = 2.10, *p* = .35). The CES-D also provides cutoff scores of 16 or greater, which aid in identifying individuals at risk for clinical depression. Of our study participants, 24 participants had scores higher than the cutoff scores.

### Apparatus and stimuli

The stimuli were presented on a 27-inch monitor (resolution of 1920×1080; refresh rate = 144 Hz, ProLite G2773HS; Iiyama Corp.). The experiment was conducted on a PC using software (MATLAB; The MathWorks Inc., Natick, MA) with Psychtoolbox [58–60].

From images presented by Konkle, Brady, Alvarez, and Oliva [61], 140 colored landscape images were chosen. All stimuli were 200 × 200 pixels. The participants’ viewing distance was approximately 58 cm. The single stimulus visual angle was 11°×11°; that for paired stimuli was 11°×18°. Stimuli were presented in the center of the screen on a black background.

### Procedure

Before the experimental task, participants completed questionnaires related to depressive symptoms. The CES-D score was used to evaluate levels of self-reported depressive symptoms.

**Fig 2:**
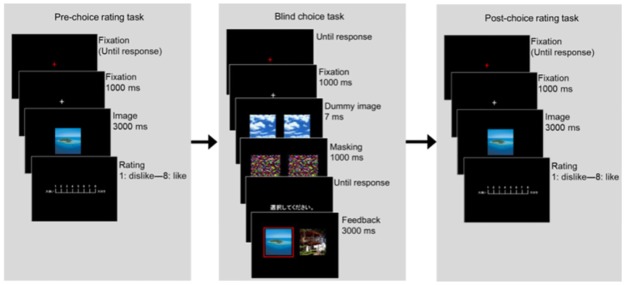
Experimental tasks. Experimental tasks constructed in three sessions: Pre-choice rating task, Blind choice task, and Post-choice rating task. In the Pre-choice rating task, participants were presented items for 3 s. They rated their preference for it on an eight-point scale. In the blind choice task, participants were instructed that two images that were rated in the pre-choice rating task were presented subliminally. Of the two images, they were asked to choose the one which they preferred. In fact, however, two identical dummy images were presented for 7 ms. After the choices were made, the two images were presented. The red rectangle denotes that it was chosen blindly. In the post-choice rating task, participants were presented the same image stimuli as those of the pre-choice rating task. Their preference was rated again.

After completing the questionnaire, participants performed three experimental tasks (a pre-choice rating task, blind choice task, and post-choice rating task, see Fig 2). The procedures of these three tasks resembled those described by Nakamura and Kawabata [53] and by Sharot et al. [54], and also our earlier EEG experiment [55]. To familiarize participants with the pre-choice rating task and blind choice task, they were given three practice trials.

For the pre-choice rating task, participants were instructed to evaluate their subjective preference for an image on screen. They were rated using an eight-point scale (1 = dislike 8 = like) on number pad buttons of a keyboard. The image was presented for 3 s on the screen. No time limit was applied for the ratings. Participants performed the ratings for the 140 images. The order was randomized across participants.

In the blind choice task, participants were told that this experiment was conducted to investigate the implicit preference judgment process. Participants were instructed that two images that were visible in the pre-choice rating task would be presented for approximately 7 ms in each trial. In fact, however, the two dummy images were presented subliminally. After the two dummy images on the right and the left sides of the screen were presented, the two masking stimuli were presented for 1 s. The masking stimuli were followed by the words “choose” on the screen, which asks participants to judge which images they prefer by pressing the keyboard keys. The dummy images were presented. Therefore, participants’ judgments could not be guided by their own preferences. However, participants were told that “it is well known that people can judge the preference intuitively for subliminally presented stimuli.” After participants made a choice, the feedback (i.e., the pair of images) presented in the pre-choice rating task was presented for 3 s along with the red rectangle which surrounds one of these images indicating a blindly chosen image. Two conditions were used during the blind choice task: a critical condition and a non-critical condition. In the critical condition (70% of 70 trials), the two images with the same pre-choice rating score were presented in the feedback. In this condition, the chosen image was determined randomly. It was presented with a red rectangle on the same side as the participant’s pressed key. In the non-critical condition (30% of 70 trials), two images with different pre-choice rating scores were presented in the feedback. In the non-critical condition, the highly rated image was always marked as the chosen image, and was presented on the same side as the participant’s pressed key. This condition was used to induce participants to believe that they were able to judge their preference intuitively, even for subliminally presented stimuli.

After the blind choice task, participants were again asked to evaluate subjective preferences for the 140 images. The post-choice rating task procedure was the same as that of the pre-choice rating task.

### Data analysis

We computed the mean-corrected ratings to analyze the choice-induced preference change by following procedures described by Sharot et al. [54]. For each pre-choice and post-choice rating session, mean-corrected ratings were calculated by subtracting the average ratings of the respective sessions from the rating of each stimulus: the mean-corrected ratings represent the relative values of respective stimuli within each session. For each participant, the preference changes after the blind choice were calculated by subtracting the mean-corrected pre-choice rating from the mean-corrected post-choice rating. Furthermore, the mean preference change score was calculated for chosen and rejected images in the blind choice task. The non-critical condition was excluded from these analyses because the change of ratings from pre-choice to post-choice is confounded by the statistical regression effect. To examine the choice-induced preference change, we used data related to a change in preference ratings, except for data from our EEG experiment, and from 13 participants who did not believe the deception of the blind choice task used for this study.

To examine correlation between a change in preference ratings and depressive symptoms, we calculated Spearman’s rank correlation coefficient to reduce the effect of outliers for CES-D scores. For correlation analysis, we included data obtained from our previous EEG experiment. Therefore, the valid data for the correlation analysis were *n* = 69 (see Fig 1).

## Results

### Choice-induced preference change on the blind choice

**Fig 3:**
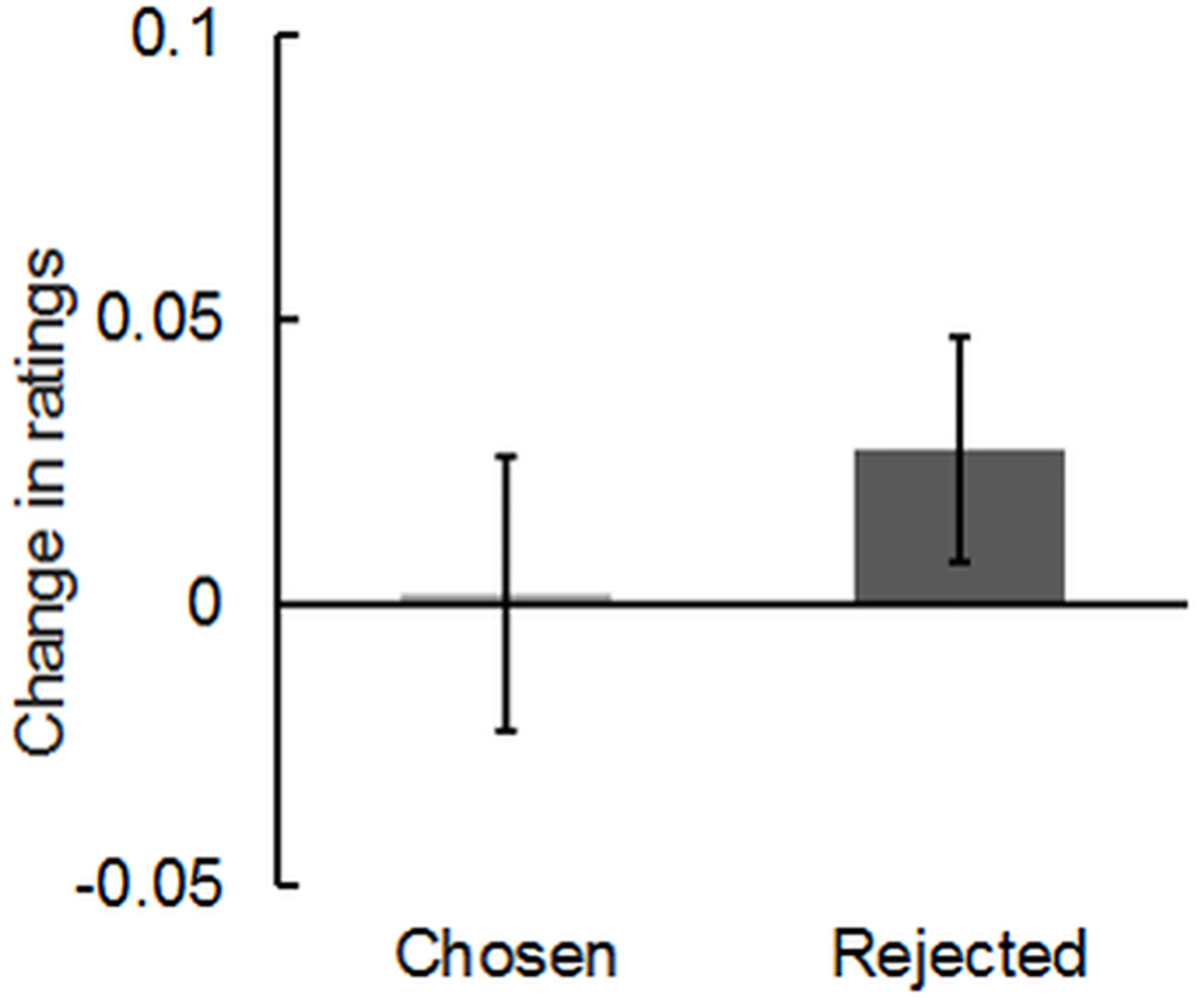
Post-choice change in preference. Change in preference ratings for chosen and rejected images of a landscape after blind choice between two equally preferred images. Bars represent differences in mean-corrected ratings between the pre-choice rating task and post-choice rating task. Error bars represent standard errors of the mean.

Fig 3 presents the mean change in ratings for chosen and rejected images on the blind choice task. To verify whether the choice-induced preference change was observed, we applied a paired *t*-test to assess differences in preference change between chosen images and rejected images. No significant difference was found between the chosen and rejected items in the change in preference ratings (*t*(43) = -0.50, *p* = .62). Additionally, we conducted one-sample *t* tests to ascertain whether each change in ratings of chosen and rejected images was significantly different from zero. Results show no significant change either for chosen images or rejected images (chosen images: *t*(43) = 0.07, *p* = .95; rejected images: *t*(43) = 1.10, *p* = .28). Actually, those results were not surprising because the effect size of choice-induced preference change is small [52].

### Correlation between choice-induced preference change and depressive symptoms

**Fig 4:**
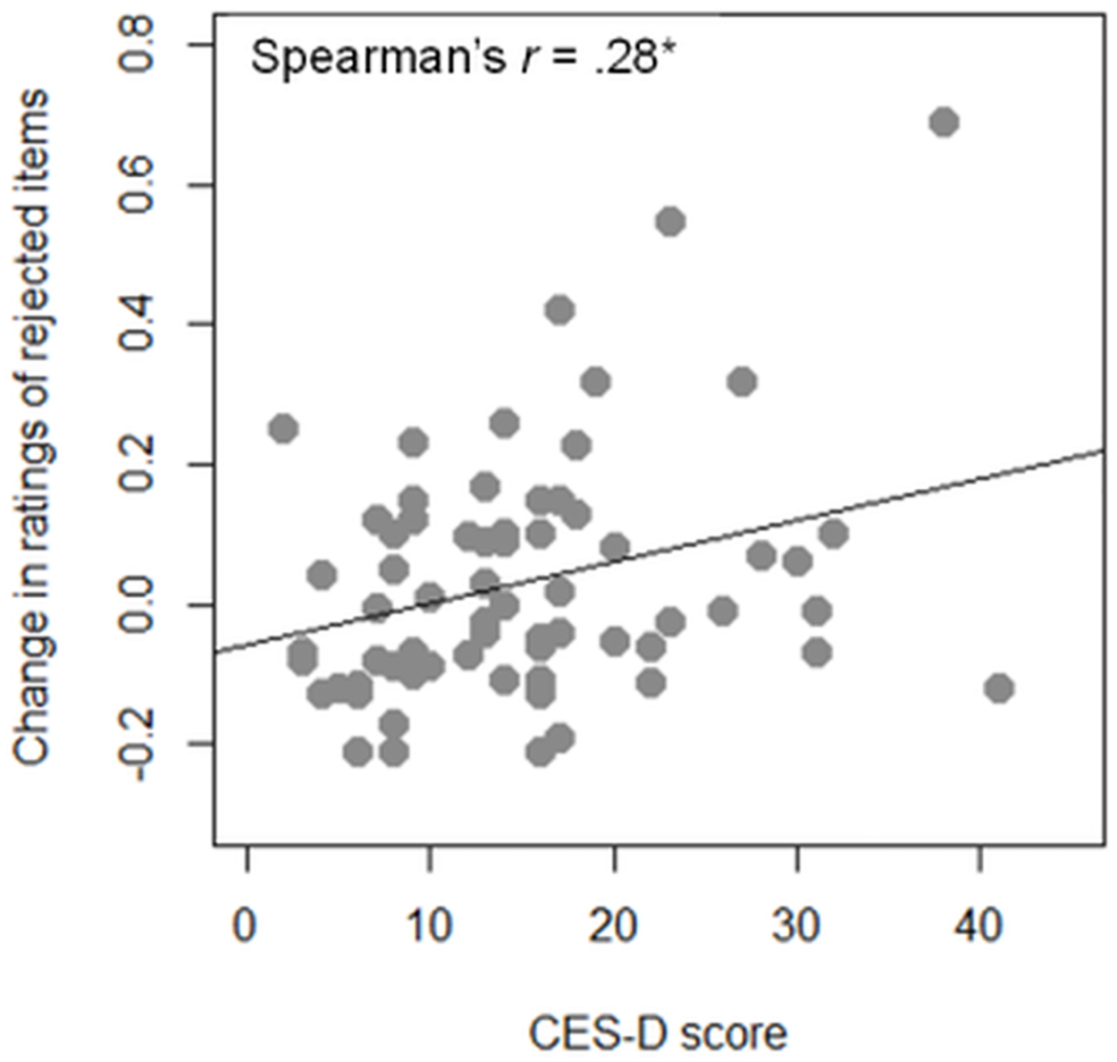
Depressive symptoms and change in preference of rejected items. Scatter plot of depressive symptoms (measured by CES-D) and change in ratings of rejected items.

Although no significant choice-induced preference change was found for the entire group of participants as a whole, significant correlation was found between choice-induced preference change and depressive symptoms. To assess the relation between choice-induced preference change and depression, we conducted Spearman’s rank correlation analysis. We corrected *p*-values using the FDR method to prevent an increase in the risk rate by multiple comparison. Results show that CES-D scores were positivity correlated with the change in ratings of rejected images (Spearman’s *r* = .28, *p* = .04; Fig 4). Results of post hoc analysis show the statistical power of this test as 73%. However, the change in the ratings of chosen images does not indicate significant correlation with depressive symptoms (Spearman’s *r* = -.02, *p* = .86). In addition, to explore sex-related differences of correlation between depressive symptoms and choice-induced preference change, we conducted partial correlation analysis when controlling for sex. Results show that significant correlation between CES-D scores and change in ratings of rejected items remains when controlling for sex (Spearman’s *r* = .27, *p* = .03).

These results demonstrate that depressive symptoms are related to choice-induced preference change. Specifically, individuals with strongly depressive symptoms tend to show no reduced preference for rejected items.

## Discussion

This study was conducted to investigate the relation between depression and choice-induced preference change. In this study, we observed no choice-induced change using the blind choice paradigm. However, as we had expected, results show that the degree of choice-induced preference change for a rejected item varies according to individual differences of depressive symptoms. Depressive symptoms in non-clinically diagnosed participants tend to show less decreased preference of rejected items. This correlation was persistent after controlling for sex. These results, which indicate that individual differences strongly affect the occurrence of choice-induced preference change, are the first ever reported demonstrating that depressive symptoms are associated with choice-induced preference change. Based on results of their meta-analysis, Izuma and Murayama [52] reported that the effect size of choice-induced preference change was extremely small. One reason is that, as demonstrated in the present study, individual differences such as depressive symptoms affect choice-induced preference change. Future studies should investigate individual differences of choice-induced preference change.

Results show that depressive symptoms are related to the preference change of rejected items, but no significant relation with depressive symptoms was found for the chosen items. These results suggest that depressive symptoms are related selectively to updating of the preference of rejected items, but they are not related to updating of the preference of chosen items. Although respective underlying processes of the preference change in chosen and rejected items remain unclear at this stage, our results underscore the need for individual explanations for the learning processes of chosen and rejected items. Choice-induced preference change is explained traditionally by cognitive dissonance theory [62] and self-perception theory [63,64]. These theories have not elucidated the factors causing the respective preference change for chosen and rejected items. According to cognitive dissonance theory, choosing one item from two equally preferred items causes aversive feelings (i.e., cognitive dissonance). After dissonance is aroused, it is resolved by justifying the original choice i.e., increase in preference for the chosen item, with a concomitant decrease in preference for rejected items. Regarding self-perception theory [63,64], when making a choice between two equally attractive items, participants learn their preferences by observing their own choices. Results show that chosen items are more preferred and non-chosen items are less preferred. These theories suggest that the same factor (i.e., cognitive dissonance or observing own overt behavior) affects symmetric preference change for chosen and rejected items. Therefore, it seems difficult to explain our result that a different relation between depressive symptoms and preference change of chosen and rejected items is based on these theories.

Can the RL theory explain our results? Although different factors related to depression (i.e., hyposensitivity to reward and hypersensitivity to loss) have been known to affect the increasing and the decreasing item values, the present results cannot be explained fully based on notions about RL theory examined in studies of externally guided decision-making. In studies of the relation between depression and externally guided decision-making, depressed individuals are known to show impaired ability to increase the item value through RL in decision-making tasks with reward-maximization goals [6,10], but they show a persistent ability to decrease the item value in the task with a loss-minimization goal [7]. The cause of the impaired ability to increase the item value is probably hyposensitivity to gain in depressed individuals [5,19], whereas the cause of retained ability to decrease the item value is probably hypersensitivity to loss [2,17]. Although those factors can apparently account for the asymmetric relation between depression and choice-induced preference change, the present results are inconsistent with these notions. Our study demonstrated that depressed participants in a non-clinical sample show retained ability to increase the preference of chosen items and impaired ability to decrease the preference for rejected items. This inconsistency might have emerged from the difference of decision-making types.

The difference between internally guided decision-making and externally guided decision-making is that the correct answer is determined by one’s own criteria or external circumstances [12,13]. In internally guided decision-making, increased self-relatedness for a chosen item is thought to function as positive reinforcement based on evidence that the reward-related neural substrates (i.e., ventral tegmental area, ventral striatum, and ventromedial prefrontal cortex) are activated by self-relevant stimuli [65–67]. Indeed, neuroimaging studies using the free-choice paradigm demonstrated that those reward-related areas were engaged for choice-induced preference change [40,46,47]. From the present data, no relation between depressive symptoms and the change in ratings of chosen items was found in depressed participants in a non-clinical sample. Therefore, the reactivity of the reward-related areas via self-relatedness might be retained in depressed participants in a non-clinical sample, different from the case of externally guided decision making (i.e., hyposensitivity to externally delivered rewards).

In contrast, the decrease of self-relatedness for the rejected item is thought to function as devaluation of the item. Consistent with this assumption, earlier studies using the free-choice paradigm demonstrated that striatal activity for rejected items is lower [40]. In the present study, no decreased preference of rejected items was found in depressed participants in a non-clinical sample. Therefore, it is possible that depressed participants in a non-clinical sample had difficulty decreasing the self-relatedness of rejected items. Given the evidence that depressed individuals feel high self-relatedness for negative stimuli [68,69], they might restore the value of the rejected item once the value for the rejected item is decreased because they feel high self-relatedness for negative stimuli (i.e., devalued rejected item). This feeling might result in the procrastinations associated with depression, by which depressed individuals tend to delay making decisions related to important matters [70–72]. Depressed individuals might tend to avoid the decreasing likelihood of choosing the rejected item in future opportunities.

In this manner, an RL-like mechanism might engage in internally guided decision-making, but the value-updating process in internally guided decision-making cannot be explained simply using the RL theory, as externally guided decision-making. The characteristics of the task and reinforcer in internally guided decision-making differ from those in externally guided decision-making. Although it is unclear whether self-relatedness for chosen and rejected items mediates RL as we have discussed above, it is possible that investigating the mechanism behind the relation between the depression and choice-induced preference change is one means of clarifying how the choice-induced preference change emerged.

What is the implication for depression? The results of the present study showing that depressed individuals show less updating of the value of rejected items are expected to provide clues to elucidate the mechanism behind the indecision of depressed individuals. Results of prior studies suggest that procrastination is associated with depression, and that depressed individuals tend to delay making decisions related to important matters [70–72]. Based on the results of the present study, because depressed individuals cannot decrease the value of rejected items, it is likely that the depressed individuals cannot clarify their own internal decision criteria. As a result, depressed individuals might tend to delay decision-making. Further choice-induced preference change studies of major depressed disorder patients are expected to provide clearer evidence for the mechanism behind the indecision and procrastination of depressed individuals.

## Limitations

Despite the importance of our data for revealing the relation between depression and choice-induced preference change, several limitations of this study must be considered. First, our study was conducted with the participation of nonclinical undergraduate and graduate students. Although our use of the CES-D points to examination of depressed participants in a non-clinical sample, it is possible that the mechanisms of preference updating in nonclinical depressed individuals and in major depressive disorder patients differ. Future research must be undertaken to elucidate the association with choice-induced preference change in major depressive disorder patients.

Secondly, although we examined the relation between choice-induced preference change and RL theory, the reinforcement process of chosen and rejected items remains unclear. Our data imply that reinforcement processes in internally guided decision-making differ from those of externally guided decision-making, and that this difference derives from characteristics of the task and reinforcer. Future studies must be conducted to explore how the RL theory framework is applicable to internally guided decision-making.

## Conclusions

This study investigated the relation between depression and internally guided decision-making. Our results demonstrated for the first time ever reported that depressive symptoms only affect preference updating of rejected items. They do not affect the preference updating of chosen items. Although the underlying processes of preference updating in chosen and rejected items remains unclear, it is possible that depression affects choice-induced preference change just as it affects externally guided decision-making. However, because of the differences in characteristics of these two types of decision-making, updating value processes in internally guided decision-making might not be explained simply and solely by RL theory, unlike externally guided decision-making. To elucidate the mechanism underlying the relation between depression and choice-induced preference change, future studies must be conducted to investigate the reinforcement process, with consideration of the characteristics of internally guided decision-making.

## Supporting information

S1 File Miyagi et al_supplement data.xlsx. (XLSX)
